# Standardized curcumin formulations in type 2 diabetes mellitus: a precision adjunct strategy targeting bioavailability, glycemic control, and immunometabolic inflammation

**DOI:** 10.3389/fnut.2026.1874887

**Published:** 2026-07-14

**Authors:** Eleje O. Okonta, Charles O. Nnadi, Ugwu Okechukwu Paul-Chima

**Affiliations:** 1 Department of Pharmacognosy and Environmental Medicine, Faculty of Pharmaceutical Sciences, University of Nigeria Nsukka, Nsukka, Nigeria; 2 Department of Pharmaceutical and Medicinal Chemistry, Faculty of Pharmaceutical Sciences, University of Nigeria Nsukka, Nsukka, Nigeria; 3 Department of Research and Publication, Kampala International University, Kampala, Uganda

**Keywords:** adjunct therapy, bioavailability, *Curcuma longa*, curcumin, glycemic control, immunometabolism, inflammation, precision nutrition

## Abstract

Type 2 diabetes mellitus is increasingly conceptualized as an immunometabolic disorder in which hyperglycemia, insulin resistance, oxidative stress, and chronic low-grade inflammation interact synergistically to accelerate vascular and end-organ complications. Curcumin, the principal polyphenolic constituent of *Curcuma longa*, has attracted sustained scientific interest as an adjunct intervention given its putative effects on glycemic regulation, inflammatory signaling, and redox homeostasis. Clinical translation has been constrained by poor aqueous solubility, limited gastrointestinal absorption, rapid phase II metabolism, and inconsistent formulation quality across investigational preparations limitations extensively documented across pharmacokinetic studies and clinical reviews. This Perspective contends that curcumin must be evaluated not as a generic botanical supplement but as a standardized, formulation-dependent adjunct requiring precision in dose selection, delivery architecture, bioavailability enhancement, biomarker specification, and patient phenotyping. Emerging platforms, including curcuminoid-piperine co-formulations, phospholipid complexes, polymeric nanoparticles, and micellar systems, may substantially improve systemic exposure and therapeutic reproducibility. Evidence from randomized controlled trials and meta-analyses indicates potential improvements in fasting plasma glucose, glycated hemoglobin, insulin resistance indices, and inflammatory biomarkers, although heterogeneity in formulation composition, intervention duration, dosing strategy, and background pharmacotherapy attenuates certainty of effect. A precision adjunct framework is proposed to identify the patient subgroups, formulation classes, and inflammatory phenotypes most likely to derive clinical benefit. Future trials should prioritize standardized preparations, pharmacokinetic validation, stratified enrolment, clinically meaningful end-points, systematic safety monitoring, and rigorous integration with guideline-concordant diabetes management.

## Introduction

Type 2 diabetes mellitus is a progressive cardiometabolic disorder characterized by insulin resistance, incremental beta-cell failure, and sustained hyperglycemia. The pathophysiology extends well beyond disordered glucose homeostasis: chronic low-grade inflammation, systemic oxidative stress, endothelial dysfunction, and aberrant adipose-immune crosstalk collectively accelerate both microvascular and macrovascular sequelae, establishing the disease as a paradigmatic immunometabolic condition. Globally, the burden of type 2 diabetes continues to expand, and the persistence of residual metabolic and inflammatory risk despite pharmacological management underscores the urgent need for complementary therapeutic strategies grounded in robust biological rationale.

Curcumin, the principal bioactive polyphenol of *Curcuma longa*, has been extensively investigated for its anti-inflammatory, antioxidant, and insulin-sensitizing properties. Proposed mechanisms encompass modulation of nuclear factor-kappa B (NF-κB), tumor necrosis factor-alpha (TNF-αα), interleukin-6 (IL-6), C-reactive protein (CRP), reactive oxygen species pathways, and downstream metabolic networks that regulate insulin signal transduction (1, 2). Despite a compelling preclinical rationale, the clinical translation of native curcumin has been substantially impeded by its poor oral bioavailability; the extent to which formulation technology modifies absorption, systemic exposure, and therapeutic performance has consequently become a central issue in translational research (1–7).

Critically, the published clinical literature on curcumin in type 2 diabetes is characterized by wide heterogeneity in formulation type, dose, treatment duration, and participant phenotype. Aggregate meta-analytic evidence suggests potential benefit for glycemic and inflammatory outcomes, yet confidence in effect estimates remains limited by these sources of variation (3–7). This Perspective argues that standardized curcumin formulations should be repositioned as precision adjuncts within the management of type 2 diabetes rather than as non-specific nutritional supplements. The central thesis is that the clinical relevance of curcumin depends on congruence between formulation technology, achieved bioavailability, administered dose, treatment duration, patient phenotype, and quantifiable glycemic and inflammatory outcomes.

## From botanical supplement to precision adjunct therapy

Conventional trial paradigms have frequently evaluated curcumin as a unitary intervention, disregarding substantive pharmacological differences between native curcumin powder, purified curcuminoid extracts, curcuminoid-piperine co-formulations, phospholipid complexes, polymeric nanocurcumin, micellar preparations, and other enhanced-delivery systems. The conflation of these pharmacologically distinct entities obscures formulation-specific effects and is a primary driver of the heterogeneity observed across clinical trials. Interpreting pooled effect estimates in the absence of formulation stratification risks either dismissing genuinely active preparations or overestimating the efficacy of formulations with negligible systemic exposure.

A precision adjunct framework reconceptualises curcumin as a formulation-dependent pharmacological entity. Within this model, curcumin does not substitute for established antidiabetic pharmacotherapy whether metformin, sodium-glucose cotransporter-2 (SGLT2) inhibitors, glucagon-like peptide-1 (GLP-1) receptor agonists, or insulin but rather complements standard treatment by targeting residual inflammatory burden, oxidative stress, and metabolic dysregulation that persist despite conventional management. The theoretical foundation for this positioning is strong: the immunometabolic substrate of type 2 diabetes provides multiple convergent targets for curcumin’s pleiotropic pharmacology, and standard antidiabetic agents do not comprehensively address inflammatory or oxidative pathways.

Meta-analytic synthesis from multiple independent groups indicates that curcumin supplementation may confer improvements in glycemic indices among individuals with type 2 diabetes. A systematic review and GRADE-assessed dose-response meta-analysis reported clinically relevant reductions in body weight and waist circumference among subjects with prediabetes and type 2 diabetes receiving turmeric or curcumin (6). A further dose-response meta-analysis confirmed reductions in HbA1c and fasting plasma glucose across a range of doses, with non-linear dose-response relationships suggesting an optimal therapeutic window (4). Earlier pooled analyses similarly documented improvements in glycemic and inflammatory markers, though confidence in effect-size estimates was moderated by considerable variability in trial design, formulation type, and intervention duration (3, 5, 7).

## Bioavailability as the central translational barrier

Bioavailability constitutes the principal pharmacological limitation of curcumin therapy, and this claim warrants substantive elaboration given its centrality to the precision adjunct argument. Native curcumin exhibits extremely poor aqueous solubility (approximately 11 ng/mL at physiological pH), undergoes extensive first-pass metabolism via intestinal and hepatic glucuronidation and sulphation, achieves only low and transient plasma concentrations following oral administration (typically below 10 ng/mL after standard 1–2 g doses of native powder), and is subject to rapid enterocytic efflux. Collectively, these physicochemical and metabolic properties produce an absolute oral bioavailability estimated at less than 1% in most preclinical models (1, 2). The therapeutic effect of any given preparation is consequently determined less by its nominal label dose and more by its capacity to enhance absorption, preserve structural integrity within the gastrointestinal milieu, and sustain tissue exposure at pharmacologically relevant concentrations.

The clinical implications of this pharmacokinetic deficit are substantial. Trials employing native curcumin powder, even at doses of 6–8 g per day, may achieve insufficient systemic concentrations to exert meaningful anti-inflammatory or insulin-sensitizing effects at target tissues. This explains the inconsistency of outcomes across trials that do not account for formulation-specific bioavailability and underscores why pooled effect estimates from heterogeneous formulation types are inherently difficult to interpret. Without confirmed systemic exposure data, it is impossible to distinguish between a genuinely inactive intervention and an inadequately delivered active compound a distinction of fundamental importance for both trial design and clinical application (1, 2).

**FIGURE 1:**
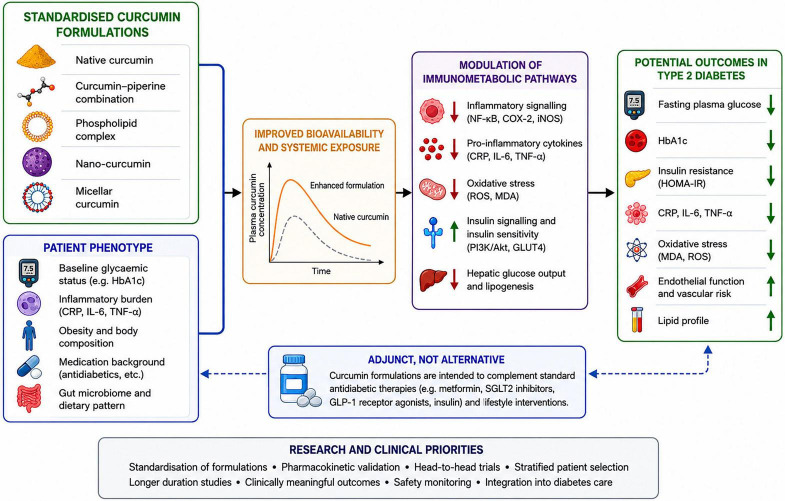
Conceptual framework for standardized curcumin formulations in type 2 diabetes mellitus. Standardized curcumin formulations may influence disease outcomes through improved bioavailability, enhanced systemic exposure, and modulation of immunometabolic signaling pathways. Patient phenotype encompassing baseline glycemic status, inflammatory burden, adiposity, medication background, and gut metabolic environment is proposed to determine therapeutic response. Enhanced delivery systems may produce effects on fasting plasma glucose, HbA1c, insulin resistance, CRP, IL-6, TNF-α, and oxidative stress biomarkers. Current evidence supports curcumin as a possible adjunct strategy rather than a replacement for standard antidiabetic pharmacotherapy.

Several formulation strategies have been developed to address these pharmacokinetic limitations, each targeting a distinct barrier to absorption and systemic exposure. Piperine co-administration inhibits phase II glucuronidation and sulphation via competitive enzyme inhibition, thereby augmenting systemic curcumin exposure by an estimated 2- to 20-fold in human pharmacokinetic studies, although the magnitude of enhancement varies with dose and timing (1). Phospholipid complexation improves membrane compatibility and facilitates lymphatic uptake by forming amphiphilic complexes that bypass enterocytic efflux and enhance transcellular permeation. Polymeric nanoparticles and micellar architectures enhance aqueous dispersibility and mucosal absorption by reducing particle size to the nanometre range, thereby increasing surface area and contact time with intestinal epithelium, whilst lipid-based delivery systems exploit bile-mediated solubilization to improve intestinal bioavailability (Figure 1).

These approaches are pharmacologically distinct in their mechanisms of action, magnitude of bioavailability enhancement, and potential for drug interactions, and should not be treated as interchangeable in either trial design or clinical application (Table 1). Reviews of formulation science confirm that newer delivery platforms can meaningfully improve solubility and bioavailability relative to native curcumin, although direct comparative clinical validation across systems including pharmacokinetic sub-studies embedded within metabolic trials remains incomplete (1, 8, 9). A further unresolved challenge is the absence of harmonized pharmacokinetic endpoints in curcumin trials, meaning that apparent clinical equivalence across formulations may reflect either genuine parity in target-tissue exposure or simply the absence of measurement.

**TABLE 1:** Potential design features of standardized curcumin formulations for type 2 diabetes mellitus.

Platform	Primary mechanism	Proposed application	Key translational challenge	Evidence context and clinical notes
Native curcumin	Basic polyphenol delivery	General supplementation	Poor absorption; negligible systemic exposure	Limited to *in vitro* and preclinical investigation; not recommended as sole agent in clinical trials
Purified curcuminoids	Standardized active-compound content	Controlled clinical investigation	Variable inter-product formulation quality	Underpins most published RCT evidence; heterogeneous outcomes attributable partly to batch variation
Curcuminoid–piperine complex	Inhibition of phase II glucuronidation	Glycemic and inflammatory modulation	Potential pharmacokinetic drug interactions with CYP450 substrates, anticoagulants	Most widely evaluated enhanced formulation; piperine augments exposure 2-20-fold but interaction risk requires monitoring
Phospholipid complex	Enhanced membrane compatibility and lymphatic uptake	Prolonged systemic exposure	Cost and cross-batch formulation variability	Phytosome technology; improves lipophilic absorption; validated in NAFLD and metabolic syndrome trials
Polymeric nanocurcumin	Improved aqueous dispersibility and mucosal absorption	Metabolic and anti-inflammatory outcomes	Comparative efficacy and long-term safety validation outstanding	Nano-curcumin in T2DM: improved FPG, HbA1c, lipids, and nesfatin versus placebo (8)
Micellar curcumin	Increased aqueous solubility via surfactant encapsulation	High-bioavailability clinical applications	Absence of head-to-head comparative trials	Highest relative bioavailability among platforms; limited T2DM-specific RCT data
Biomarker-guided dosing	Alignment of treatment with inflammatory phenotype	Precision adjunct therapy	Validated responder profiles not yet established	CRP, IL-6, TNF-α, HOMA-IR proposed as stratification and response biomarkers
Combination with standard care	Complementary targeting of residual inflammation	Adjunct to antidiabetic pharmacotherapy	Risk of therapeutic overclaiming without robust evidence	Consistent with precision adjunct framework; curcumin does not substitute for metformin, SGLT2i, or GLP-1 RA

CRP, C-reactive protein; FPG, fasting plasma glucose; GLP-1 RA, glucagon-like peptide-1 receptor agonist; HbA1c, glycated hemoglobin; HOMA-IR, homeostatic model assessment of insulin resistance; IL-6, interleukin-6; NAFLD, non-alcoholic fatty liver disease; NF-κB, nuclear factor-kappa B; RCT, randomized controlled trial; SGLT2i, sodium-glucose cotransporter-2 inhibitor; T2DM, type 2 diabetes mellitus; TNF-α, tumor necrosis factor-alpha.

## Glycemic control and metabolic outcomes

Curcumin may modulate glycemic control through several converging mechanisms. At the molecular level, inhibition of NF-κB and downstream pro-inflammatory signaling reduces serine phosphorylation of insulin receptor substrate-1 (IRS-1), thereby restoring insulin signal transduction through the PI3K/Akt/GLUT4 axis. Attenuation of hepatic gluconeogenesis through suppression of phosphoenolpyruvate carboxykinase (PEPCK) and glucose-6-phosphatase (G6Pase) activity represents a further mechanism, complementing the anti-inflammatory effects with a direct impact on fasting hepatic glucose output. Reduction of oxidative stress through induction of Nrf2/HO-1 signaling and scavenging of reactive oxygen species may additionally ameliorate glucotoxicity-mediated impairment of beta-cell function and peripheral insulin sensitivity. Favorable modification of adipokine profiles including reductions in leptin and increases in adiponectin may further support metabolic improvement. Clinical investigations have principally evaluated fasting plasma glucose, glycated hemoglobin (HbA1c), fasting insulin, and the homeostatic model assessment of insulin resistance (HOMA-IR) as primary metabolic end-points.

The aggregate evidence from randomized controlled trials and meta-analyses indicates potential benefit for fasting glucose, HbA1c, and indices of insulin resistance, though findings are not uniform across studies (3, 7, 10). Altobelli et al. (3) conducted a meta-analysis of RCTs specifically in uncomplicated type 2 diabetes and reported statistically significant reductions in HbA1c and fasting blood glucose with curcumin supplementation versus placebo. The dose-response analysis of Bahari et al. (4) similarly identified a non-linear relationship between curcumin dose and glycemic outcomes, with evidence that moderate doses (500–1,500 mg per day of bioavailable formulations) may achieve greater glycemic reduction than higher doses of poorly bioavailable preparations a finding with direct implications for clinical dose selection. Moradi Baniasadi et al. (6) extended these observations to anthropometric outcomes, reporting significant reductions in body mass index and waist circumference in addition to glycemic indices, suggesting that curcumin’s metabolic effects may extend beyond direct insulin sensitization to encompass adiposity-related mechanisms.

Residual heterogeneity across trials is attributable to differences in baseline glycemic status, concomitant pharmacotherapy, intervention duration, administered dose, and the pharmacokinetic profile of the specific formulation employed. A precision stratification approach would enroll participants according to baseline HbA1c strata, degree of insulin resistance, adiposity, systemic inflammatory burden, background medication class, and verified curcumin plasma concentration, substantially improving the interpretability and translational relevance of trial outcomes.

## Immunometabolic inflammation

The chronic inflammatory substrate of type 2 diabetes provides a particularly strong mechanistic rationale for curcumin investigation. Persistently elevated concentrations of CRP, TNF-α, and IL-6 contribute directly to insulin resistance through serine phosphorylation of IRS-1, impair vascular endothelial function through suppression of endothelial nitric oxide synthase (eNOS) activity, and accelerate the progression of macrovascular disease through promotion of atherogenic lipid modification, foam cell formation, and plaque instability. Adipose tissue macrophage infiltration and polarization toward the pro-inflammatory M1 phenotype further amplify systemic cytokine production in the context of central obesity, creating a self-reinforcing cycle of adipose dysfunction, systemic inflammation, and metabolic deterioration.

Curcumin’s distinctive value in this context lies in its capacity to act at the convergence of metabolic and inflammatory signaling networks, potentially interrupting pathological feedback loops that perpetuate both hyperglycemia and vascular injury. The principal anti-inflammatory mechanisms include inhibition of IκB kinase (IKK), which prevents nuclear translocation of NF-κB and downstream transcription of pro-inflammatory cytokines; suppression of cyclooxygenase-2 (COX-2) and inducible nitric oxide synthase (iNOS); and activation of Nrf2-mediated antioxidant gene expression. Together, these actions position curcumin as a multi-target immunometabolic modulator rather than a selective anti-inflammatory agent.

Quantitative syntheses of randomized controlled trial data indicate that curcumin supplementation may reduce circulating concentrations of CRP, TNF-α, IL-6, and the oxidative stress marker malondialdehyde (MDA), though the magnitude and consistency of these effects depend on trial-level characteristics and population heterogeneity (5, 7). Mokgalaboni et al. (5) reported statistically significant reductions in both glycemic indices and inflammatory markers in their quantitative meta-analysis of RCTs, reinforcing the dual metabolic and immunological rationale for curcumin supplementation in type 2 diabetes. This evidence collectively supports the characterization of curcumin as an immunometabolic adjunct an agent whose principal clinical value lies in addressing the inflammatory and oxidative dimensions of type 2 diabetes that persist despite conventional glycemic management.

## Precision nutrition and patient stratification

A critical methodological limitation of the extant curcumin literature is the implicit assumption that all individuals with type 2 diabetes carry an equivalent probability of treatment response. Heterogeneity in response is likely determined by inflammatory phenotype, adiposity, gut microbiome composition, concomitant pharmacotherapy, habitual dietary pattern, disease duration, and baseline metabolic control. Failure to account for these sources of variation conflates genuinely unresponsive patients with those who receive inadequate formulation exposure or are otherwise mismatched to the intervention.

Inflammatory phenotyping is a particularly promising stratification variable. Patients with type 2 diabetes can be broadly partitioned into those with predominantly inflammatory-driven insulin resistance characterized by elevated CRP, IL-6, and TNF-α, higher visceral adiposity, and greater macrovascular risk and those whose disease is driven principally by beta-cell insufficiency with a less prominent inflammatory signature. The immunometabolic mechanisms of curcumin suggest that benefit would be concentrated in the former subgroup, and stratified trial designs testing this hypothesis would generate actionable responder profiles. Dose-response meta-analyses from Bahari et al. (4) and Moradi Baniasadi et al. (6) further suggest that response may also vary with baseline glycemic severity and adiposity, providing additional stratification dimensions worthy of prospective investigation.

Precision nutrition methodology offers a principled framework for improving both trial design and clinical interpretation. Future investigations should systematically evaluate whether curcumin’s therapeutic effects are concentrated in patient subgroups characterized by elevated CRP, marked insulin resistance, abdominal obesity, early-stage disease, or heightened oxidative stress. Such stratification would generate responder profiles applicable to clinical decision-making and would substantially improve the precision and generalizability of future meta-analytic estimates. Biomarker-guided dosing in which formulation choice and dose are determined by confirmed plasma curcumin exposure and pre-treatment inflammatory status represents the most rigorous application of this framework and should be considered a design priority for future trials.

## Safety, regulation, and clinical integration

Curcumin is generally considered well tolerated across the doses employed in clinical trials; however, safety cannot be extrapolated uniformly across all formulations, dose levels, or clinical populations. Enhanced-bioavailability systems increase systemic exposure and thereby raise the potential clinical significance of adverse pharmacodynamic effects and pharmacokinetic drug interactions. Heightened vigilance is warranted for patients receiving anticoagulant or antiplatelet therapy, those prescribed multiple glucose-lowering agents, and individuals dependent on drugs subject to hepatic cytochrome P450 (CYP3A4, CYP1A2) metabolism. Piperine-containing co-formulations merit particular caution given the broad enzyme-inhibition profile of piperine and its potential to alter the pharmacokinetics of co-administered antidiabetic, cardiovascular, and anticoagulant agents.

The regulatory status of curcumin products warrants commensurate attention. The widespread marketing of curcumin as a nutritional supplement exists in tension with the therapeutic claims increasingly advanced for enhanced-bioavailability formulations intended for use in metabolic disease a tension that demands a correspondingly higher evidentiary standard, analogous to that applied to botanical pharmaceuticals in regulated markets. Clinical integration should therefore remain conservative: curcumin may be judiciously considered as an adjunct to, rather than a substitute for, guideline-concordant antidiabetic therapy, and its use should be disclosed to and supervised by a qualified healthcare provider. Until pharmacokinetically validated, long-duration, adequately powered RCTs are available for specific enhanced-delivery formulations, clinical recommendation should be restricted to populations in whom the benefit-risk profile is most clearly favorable.

## Research priorities

Advancing this field requires a substantive departure from undifferentiated curcumin trials toward investigations designed with pharmacological precision. Several priority domains can be identified. First, direct head-to-head comparisons of rigorously standardized formulations are essential to establish the relative bioavailability, pharmacokinetic profile, and clinical performance of piperine co-formulations, phospholipid complexes, polymeric nanocurcumin, and micellar systems. Without this comparative evidence, the selection of formulation for clinical use remains empirical.

Second, pharmacokinetic sub-studies embedded within clinical trials should confirm systemic exposure at the population level, enabling *post hoc* correlation of plasma curcumin concentrations with clinical response a design feature that would substantially improve the mechanistic interpretability of outcomes. Third, intervention durations of at least 12 weeks, and ideally 24 weeks or beyond, are necessary to detect clinically meaningful change in HbA1c, given the erythrocyte lifespan upon which this biomarker depends. Fourth, sample sizes should be adequately powered for primary metabolic end-points, using effect-size estimates derived from formulation-specific rather than pooled data.

Core outcome sets should encompass HbA1c, fasting plasma glucose, HOMA-IR, CRP, IL-6, TNF-α, MDA, lipid profiles (total cholesterol, LDL-cholesterol, HDL-cholesterol, triglycerides), anthropometric indices (body mass index, waist circumference), adverse events, and any modifications to background antidiabetic pharmacotherapy. Systematic reviews and meta-analyses should routinely execute subgroup analyses stratified by formulation platform, administered dose, intervention duration, baseline HbA1c category, concomitant medication class, and pre-treatment inflammatory status. Without this level of analytical precision, pooled effect estimates will continue to obscure clinically actionable information. The recent GRADE-assessed dose-response analyses of Bahari et al. (4) and Moradi Baniasadi et al. (6) represent methodological advances in this direction and should be regarded as exemplars for future syntheses.

## Discussion

Curcumin occupies a scientifically credible yet empirically contested position within the landscape of type 2 diabetes research. The mechanistic rationale is well established across preclinical and translational models, and a growing body of randomized trial and meta-analytic evidence supports the possibility of meaningful improvements in glycemic and inflammatory outcomes in appropriately selected patient groups. Nevertheless, uncritical enthusiasm is unwarranted given the pervasive heterogeneity of existing trials, the profound dependence of therapeutic effects on formulation characteristics, and the inherent limitations of predominantly short-duration interventions in a disease defined by decades of progressive metabolic deterioration.

The evidence base has expanded substantially with the publication of recent dose-response meta-analyses (4, 6) that extend beyond earlier pooled analyses (3, 7) by explicitly modeling dose-response relationships and applying GRADE methodology to assess evidence certainty. These advances partially address the completeness limitations of earlier literature coverage but also highlight that confidence ratings for most outcomes remain low to moderate, chiefly because of risk of bias in primary trials and unexplained statistical heterogeneity. The implication is not that curcumin is ineffective but that the evidence base is not yet sufficient to support definitive clinical guidance for any specific formulation at any specific dose.

A particularly important insight emerging from the aggregate literature is the distinction between glycemic effects and anti-inflammatory effects. The evidence for reductions in CRP, IL-6, and TNF-α is, in several respects, more consistent than the evidence for HbA1c reduction, possibly reflecting the more direct mechanistic link between curcumin’s NF-κB inhibition and circulating cytokine concentrations. If this hierarchy of effect is confirmed in formulation-stratified trials, it would argue for a primary positioning of curcumin as an immunometabolic adjunct rather than a glycemic agent per se, with glycemic benefit understood as a downstream consequence of inflammation resolution and insulin sensitivity restoration.

The most scientifically defensible and clinically responsible advancement is the development of a precision adjunct framework that delineates the patient population, formulation class, dose range, intervention duration, and biomarker targets for which curcumin offers a credible therapeutic contribution. Such a framework would situate curcumin research within contemporary standards of precision nutrition and evidence-based adjunct pharmacotherapy, thereby enabling regulatory-grade evidence synthesis and the development of informed clinical practice guidance. The figure quality concerns raised during peer review also apply more broadly: future publications in this space would benefit substantially from high-resolution conceptual frameworks that clearly depict the relationships between formulation platform, pharmacokinetic exposure, patient phenotype, and clinical outcomes.

## Conclusion

Standardized curcumin formulations represent a scientifically plausible precision adjunct strategy for type 2 diabetes mellitus, particularly in the context of glycemic dysregulation accompanied by chronic low-grade inflammation. The realization of their clinical potential depends on the systematic resolution of bioavailability limitations which represent the primary pharmacological barrier to consistent therapeutic effect rigorous differentiation of formulation-specific effects, identification and validation of responder phenotypes, and the safe incorporation of curcumin within guideline-concordant diabetes management. The expanding evidence base, including recent dose-response meta-analyses incorporating GRADE methodology, strengthens the mechanistic plausibility of this approach while simultaneously underscoring the need for trials of greater pharmacological precision. Repositioning curcumin from a generic botanical supplement to an evidence-based immunometabolic adjunct anchored in standardized preparations, pharmacokinetic validation, and clinically meaningful outcome assessment is both scientifically justified and clinically imperative.

## Data Availability

The original contributions presented in this study are included in this article/supplementary material, further inquiries can be directed to the corresponding author.
